# Characterizing Word Embeddings for Zero-Shot Sensor-Based Human Activity Recognition

**DOI:** 10.3390/s19225043

**Published:** 2019-11-19

**Authors:** Moe Matsuki, Paula Lago, Sozo Inoue

**Affiliations:** 1 Department of Applied Science for Integrated System Engineering Kyushu Institute of Technology, Kitakyushu 804-8550, Japan; 2 Department of Basic Sciences Kyushu Institute of Technology, Kitakyushu 804-8550, Japan; paula@mns.kyutech.ac.jp; 3 Department of Human Intelligence Systems Kyushu Institute of Technology, Hibikino 808-0196, Japan; sozo@brain.kyutech.ac.jp

**Keywords:** human activity recognition, Zero-shot machine learning, word embedding representation

## Abstract

In this paper, we address Zero-shot learning for sensor activity recognition using word embeddings. The goal of Zero-shot learning is to estimate an unknown activity class (i.e., an activity that does not exist in a given training dataset) by learning to recognize components of activities expressed in semantic vectors. The existing zero-shot methods use mainly 2 kinds of representation as semantic vectors, attribute vector and embedding word vector. However, few zero-shot activity recognition methods based on embedding vector have been studied; especially for sensor-based activity recognition, no such studies exist, to the best of our knowledge. In this paper, we compare and thoroughly evaluate the Zero-shot method with different semantic vectors: (1) attribute vector, (2) embedding vector, and (3) expanded embedding vector and analyze their correlation to performance. Our results indicate that the performance of the three spaces is similar but the use of word embedding leads to a more efficient method, since this type of semantic vector can be generated automatically. Moreover, our suggested method achieved higher accuracy than attribute-vector methods, in cases when there exist similar information in both the given sensor data and in the semantic vector; the results of this study help select suitable classes and sensor data to build a training dataset.

## 1. Introduction

Human activity recognition using data obtained from wearable sensors is a technology necessary for ubiquitous computing [1]. This technology is particularly useful in fields such as care-giving [2] and manufacturing [3], while it also finds applications in security [4]. Most of the existing methods are based on supervised machine learning and require training data to be collected, labeled with correct activity annotations and times. The data collection and annotation task is quite laborous. Moreover, defining all possible activities within a context and collecting data for each one of them may be unreasonable as the definition of an activity is purely subjective. As an example, consider activities of daily living: defining all possible activities and collecting data for each of them may not be even feasible.

To eliminate the need for manual activity definition and data collection, zero-shot learning has been proposed [5,6,7,8,9,10]. The goal of zero-shot learning is to estimate unknown classes that do not appear in the training data. Almost all supervised learning methods require some sensor data to be collected for all classes, but zero-shot learning enables us to introduce unknown classes and omit the data collection task for them. Activity recognition with zero-shot learning methods is therefore much more efficient.

**Figure 1:**
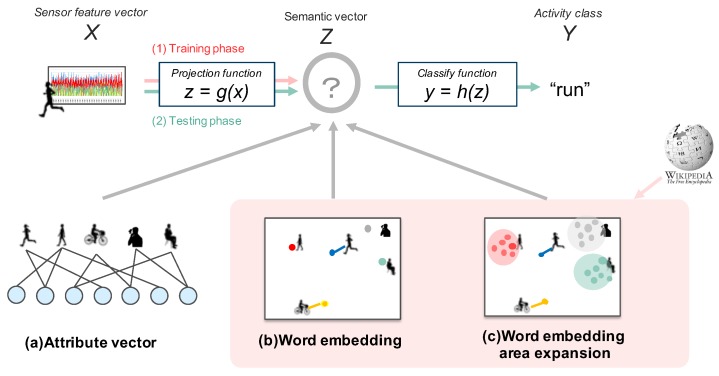
Overview of the zero-shot learning method. In the learning phase, we learn how to project feature vectors (*X*) onto semantic vectors (*Z*) from training data (red arrow). In the testing phase, we project test feature data onto the semantic space, and then identify the class (*Y*) to which the estimated semantic vector belongs (green arrow). In this paper, we compare 3 methods with different semantic spaces.

Two of the basic notions in zero-shot learning are the *feature space* and the *semantic space*. See Figure 1. The feature space contains *feature vectors*, which are obtained from sensor data. The semantic space contains *semantic vectors*, which are semantic representations of activity classes generated by human general knowledge, such as a dictionary. As an example of semantic representation, “run” can be explained as “foot moves up and down quickly, for a body to go ahead fast”. A semantic vector transforms this knowledge into a vector representation, which can be attribute-based, by manual transformation, or embedding-based, by using natural language processing techniques. Since there is one semantic vector corresponding to one class, which means classification in the semantic space is a one-to-one mapping. Therefore, in the estimation phase of zero-shot learning, a feature vector is projected into the semantic space. The resulting semantic vector is then identified as a class by searching nearest semantic vector belonging to an unknown classes. Learning known activity classes through the semantic vector space enables us to identify unknown classes by sharing semantic information from known to unknown classes.

As semantic vector, existing research uses two main types: attribute vector and word embedding vector, which are attribute vector [11] and embedding vector [5,6,7,8,9,10,12,13,14,15]. The attribute vector is generated manually by human expert knowledge either by crafting the attribute for the task or by using Wordnet. The embedding word vector is generated by natural language processing using deep networks such as Word2vec and Glove, and is widely studied for image recognition and natural language processing. We argue the embedding vector is important for activity zero-shot recognition because using attribute vectors has the following limitations: they are non-scalable and their recognition performance greatly depends on them being different for every class. In particular, they are difficult to design since the attributes of each activity are different between users. For instance, “cleaning” activity can be defined by the attribute “vaccum” for some users but perhaps for some others the activity has a different attribute, such as“mop”. However, few zero-shot activity recognition methods based on embedding vector have been studied [12,13]; especially for sensor-based activity recognition, no such studies exist, to the best of our knowledge. Although word embedding can be more efficient than attribute vectors, there are two concerns that require further study:

- Word embedding vector has meaning ambiguity and representation complexity and,
- Word embedding vector is unstable depending on corpus and learning task used.

Therefore, we want to study whether the embedding vector is useful for sensor-based zero-shot activity recognition performance, and which type of embedding (simple/expanded) is efficient for the task.

In this paper, we compare three semantic representations for zero-shot sensor activity recognition: (a) attribute vector, (b) word embedding, and (c) expanded word embedding to understand the following aspects:

1. In the embedded word vector space, are the issues of meaning ambiguity and representation complexity solved by expanding the region?
2. Do these issues affect negatively the performance of sensor-based zero-shot activity recognition?
3. Is the performance of embedded word vector increased when it closely represents the attributes defined by human?

To answer these questions, we use three data sets to evaluate each semantic representations. Our results show that using word embedding might be more efficient than using hand-generated attribute vector. Word embedding performance is slightly better than attribute vector. Moreover, there is a bigger correlation between sensor and semantic vectors when using embedding word vectors. Surprinsingly, we also find that the performance of word embedding vectors is not related to the correct meaning of words, nor to their similarity with the attribute vector. Considering these results, for sensor-based zero-shot activity recognition, the zero-shot model should choose semantic vectors considering the correlation with features of sensor data to classification.

The main contributions in this paper are summarized as follows:

- For the first time in the field of sensor-based activity recognition, we study zero-shot learning using word embedding as semantic space. In particular, we expose the difficulties of handling word embedding for human activity recognition, and study a solution via region expansion.
- We compare word embedding to the hand-crafted attribute vector in terms of accuracy of recognition of unknown classes and similarity of the spaces. We demonstrate that the automatically generated word embedding representation can perform as good as expert-designed attribute vectors.
- We examine the impact of region expansion on the performance of zero-shot recognition, as well as on the correctness of the meaning of the semantic vector. We demonstrate that there is no correlation between correct meaning and recognition accuracy. Instead, the performance depends on the correlation of the semantic space to characteristics measured by sensor data.

The organisation of the paper is as follows: we present related previous work in Section 2, while in Section 3 we introduce zero-shot learning, semantic representation, word embedding, and area expansion. In Section 4, we discuss how we evaluate the methods and we present the datasets used in the evaluation, and in Section 5 we give the results of the evaluation. Finally, we discuss our results in Section 6, and we conclude in Section 7.

## 2. Related Work

This section is organized as follows. We introduce activity recognition in Section 2.1 and zero-shot learning in Section 2.2.

### 2.1. Sensor-Based Activity Recognition

Human activity recognition has its origins in the 1990s, and the literature [16] shows that the data obtained from sensors attached to the human body provide sufficient information to detect human movements. To recognize human activity, some researches have been using video [17]. These video based activity recognition researches have ability to detect human pose, but these are not useful on private space and complicated space, such as living spaces. The sensor-based activity recognition researched can detect motion of human body parts while invading minimum privacy. So the sensor-based activity recognition has been attentioned for activity recognition for life and health care. Most of the existing methods use supervised machine learning algorithms, e.g., Bayesian methods [18], SVM [19], decision trees [20] and also DNN recently [21,22].

Although these methods can achieve high accuracy in activity classification problems, they have the shortcoming that they can estimate only activity classes that exist in the learning data. In other words, it takes time and effort to collect sensor data for all activity classes that we want to estimate. These problems pose a major obstacle when implementing an application system. For instance, implementing a monitoring system for elders requires that we collect data for each living activity. However, this task is difficult because it imposes limitations on elders while the data collection is in progress.

To tackle this problem, researchers have proposed methods using unsupervised machine learning [23] and transfer learning [24,25]. In [23], unsupervised learning for activity recognition is proposed, assuming that the number of activities *k* is unknown. However, with this method it is not possible to identify which are the classes. A different approach [25] uses the model learned from data for some users to omit the collection of data for other users. In our case, we handle the collection problem by omitting to collect data for some classes. zero-shot learning can omit to collect data for some classes by setting them as unknown classes. This method promotes activity recognition technology.

### 2.2. Zero-Shot Learning

Interest on zero-shot learning has been increasing in recent years [26]. The main idea behind zero-shot learning is to share semantic knowledge from the seen classes to the unseen classes [11]. In sensor-based activity recognition, the most used approach is using attribute vectors as a semantic space to share this knowledge. In an attribute vector, values define if the class has or doesn’t have the corresponding attribute. Each class is then represented by one vector.

However, using attribute vectors has some limitations because both the attributes and each class representation are defined manually. For this reason, attribute vectors are non-scalable: we need to define new attributes and vectors when new unknown classes appear. Moreover, recognition performance of attribute vectors greatly depends on them being different for every class. It is unreasonable to think that it is possible to define attributes for all possible classes. To overcome these problems, some methods using word embedding instead of attribute vectors as semantic space have been proposed [5,6,7,8,9,10,12,13,14,15]. To create the embeddings, unsupervised learning (such as word2vec [27,28] and Glove [29]) is used with large text corpus (such as Wikipedia). Then, the embedding vector representing the word associated with each unknown activity class is choosen for the semantic space. Using the embedding vector instead of the attribute vector, the method becomes more scalable because we can add unknown clasees by picking up new words from Wikipedia embedding vectors. Also, we don’t need to define the semantic vector manually, because it is generated automatically from the text corpus. Moreover, it is known that the methods using embedding vectors address domain problem [7]. However, as mentioned before, word embedding are created from a large, general domain text corpus, which implies that a lot of information is compacted into the embedding vector. Therefore, word embedding vectors can be complicated and ambiguous. These problems impact the accuracy of activity recognition.

Although the use of embeddings has been evaluated for object recognition in images, there are few studies [12,13,14] on their use in activity recognition. Since the words for actions (verbs) can be more complex than those for objects (noun) due to conjugations and other changes, it is necessary to separately study their performance in zero-shot activity recognition. To solve the problems mentioned above, it has been proposed to use exploding semantic vectors [13,14]. The aim of the exploding semantic vectors is making the semantic vector make general by considering the similar ebbedding vectors. An exploding semantic vector is an embedding vector calculated as the average of similar embedding vectors [14], or learned by a neural network [13]. This is similar to our expanded word embedding vector, however, the difference lies in the fact that we use similar word embedding vectors for each class as *additional* samples. Moreover, these studies are limited to video-based activity recogntion, whereas we analyze the use of embedding vectors and expanded word embedding vectors for sensor-based activity recognition. In particular, we analyze if the meaning of embedding vectors is correct with human sense by comparing them to the human-made attribute vectors and if using expanded word embedding vectors improves performance.

Previous studies for sensor-based zero shot activity recognition focus more on the use of attribute vectors [15,30,31]. However, each research generates its own set of attributes according to the dataset used, exposing one of the main shortcomings of this method. Each time, a new set of attributes has to be created, and the set of attributes defined impact the performance of the method. In this paper, we apply the methods to three datasets (simple activities, living activities, laboratory activities) and use the attribute set defined by Wang at al. [15], which were custom made for the last two datasets, to compare the attribute vectors to word embedding.

## 3. Zero-Shot Learning with Word Embedding

In this section, we explain the zero-shot learning method for activity recognition using word embedding. We begin by introducing the zero-shot learning method in Section 3.1. Details about the two stages of the method, the projection and the classification process, are given in Section 3.2 and Section 3.3, respectively. We conclude with Section 3.4, where we discuss the zero-shot method using word embedding.

### 3.1. Zero-Shot Learning Setting

We begin by explaining activity recognition with supervised machine learning methods. Let $X\subset R^{d}$ be a feature vector space, and let Y be a set of activity classes. Then,

$${\left\{\left(x_{i}^{tr},y_{i}^{tr}\right)\right\}}_{i=1}^{N^{tr}}\subseteq X\times Y$$

denotes a training dataset, where the superscript tr stands for “training” and $N^{tr}$ denotes the number of training samples.

For supervised machine learning problems, the goal is to generate the function y=f(x) that estimates ${\left\{y_{i}^{ts}\right\}}_{i=1}^{N^{ts}}\subseteq Y$ from test feature vectors ${\left\{x_{i}^{ts}\right\}}_{i=1}^{N^{ts}}\subseteq X$. It denotes a testing dataset, where the superscript ts stands for “testing” and $N^{ts}$ denotes the number of testing samples.

In zero-shot learning, we have semantic vectors in the form

$${\left\{\left(z_{i}^{tr},y_{i}^{tr}\right)\right\}}_{i=1}^{N^{tr}}\subseteq Z\times Y,$$

where $Z\subset R^{t}$ denotes a semantic vector space. The process of zero-shot learning is the same as supervised machine learning where we generate the estimation function from the training dataset, and then we estimate a class by using this function. The difference is that the test classes are unknown in the zero-shot case. In this paper, we represent unknown classes as ${\left\{y_{i}^{ts}\right\}}_{i=1}^{N^{ts}}\subseteq Y$. The zero-shot learning method estimates using two functions (projection function z=g(x) and class-output function y=h(z) by searching nearest vector). We discuss now the zero-shot learning method in detail, divided into the learning phase and the test phase.

**Figure 2:**
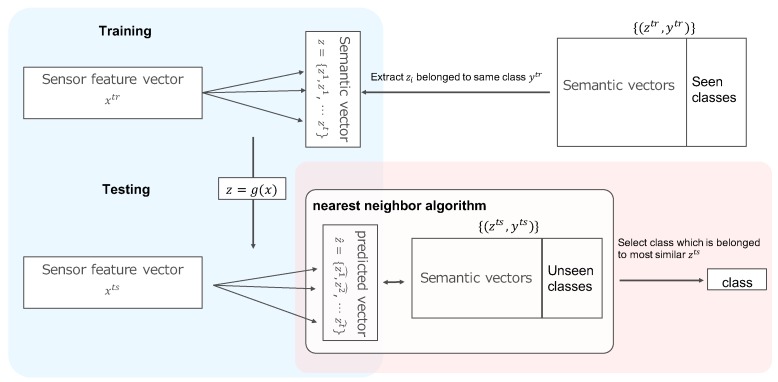
Overview of the zero-shot learning method. The top show the trainig phase and the bottom show the testing phase. The blue part shows the projection phase, and the red part shows the classification phase.

**(1)**  **Training phase (red dotted lines in Figure 2)**

The projection function z=g(x) generates a semantic vector *z* from a feature vector $x^{tr},y^{tr}$. During the learning phase, it learns each instance that belong to same class $y^{tr}$. Details about the learning phase are given in Section 3.2.

**(2)**  **Test phase (green dotted line in Figure 2)**

In the test phase, we use two functions z=g(x) and y=h(z). First, by using z=g(x), we estimate $\hat{z}$ from $x^{ts}$. Next, by using the classification function y=h(z), we estimate $\hat{y}\in Y$ from $\hat{z}$. Details about the classification function are given in Section 3.3.

Let $y^{ts}\in U$ denote a set of test classes, and let $y^{tr}\in S$ denote a set of training classes; the important point about zero-shot learning is that

U∩S=∅.

### 3.2. Projection Model

In this section we describe how to project from the feature vector space to the semantic vector space X→Z. Let $x_{i}\in N^{d}$ be a feature vector instance with *d* dimensions, and let $z_{i}\in N^{t}$ be a semantic vector instance with *t* dimensions. We can also write $z_{i}=z_{i}^{1},z_{i}^{2},z_{i}^{3},\ldots ,z_{i}^{t}$. In training phase, for each feature vector, we have an associated semantic vector. The semantic vector is associated based on the class of the feature vector. Then, each dimension of $z_{i}$ is learned from the training feature vector $x_{i}$ by using a linear regression algorithm. In this paper, we use SVR (Support Vector Regression) as regression algorithm for evaluation, and we call this method SVRBM (SVR-classifier-baser method) with SVR algorithm to project. In this process, we learn *t* regressions in one instance, one for each dimension of $z_{i}$. During the test phase, *t* real values ${\hat{z}}_{i}^{t}$ are estimated from one instance $x_{i}$, and then one semantic vector $\hat{z}$ is estimated by combining ${\hat{z}}_{i}^{t}$.

### 3.3. Class-Output Function

In this section, we explain how to estimate an unknown activity class y∈U from $\hat{z}$. We use the nearest neighbor algorithm:

$$\hat{y}=\underset{y_{ts}}{\arg\;\min}h\left(\hat{z}\right).$$

Recall that $\hat{z}$ is projected in semantic space by the projection function g(x). If we can share information between semantic vectors correctly, $\hat{z}$ is projected near the semantic vector $z_{i}$ corresponding to the class of $y_{i}$. In this case, we can successfully estimate the unknown class by using the nearest neighbor method.

### 3.4. Word Embedding and Word Embedding Area Expansion

#### 3.4.1. Word Embedding

The word embedding in this paper is a vector representation generated using word2vec, which is word vector generation tool based on unsupervised learning, using Wikipedia as corpus. The advantages of using word2vec is that (a) the word embedding can be automatically generated, (b) no identical vectors are created, and (c) the word embedding is adequate as semantic embedding space. Although existing methods use word embedding generated from word2vec as semantic vectors for image recognition, in this paper we give a first attempt to use this technique in the context of sensor activity recognition.

The word embedding generation method that we use consists in the following steps:

1. Generate a word vector $Z_{all}$ by using word2vec for all Wikipedia words.
2. Suppose that the required activity class is $Y=y_{1},y_{2},\ldots ,y_{n}$ which is including seen and unseen.
3. The set $Z_{s}=z_{1},z_{2},\ldots ,z_{n}$ corresponding to $Y=y_{1},y_{2},\ldots ,y_{n}$ is extracted from $Z_{all}$.

There are some activity classes that combine two or more words, such as “Open door”. In this case, the vectors of each word (“Open” and “door”) are averaged for each dimension.

#### 3.4.2. Expanded Word Embedding

This semantic space is generated from word embedding $Z_{s}=z_{1},z_{2},\ldots ,z_{n}$ and $Z_{all}$. For each instance $z_{n}$, we obtain *k* samples by calculating distances between semantic vectors in $Z_{all}$. The samples closest to the instance $z_{n}$ are considered as instances belonging to the class $y_{n}$. For the purpose of evaluation, the parameter *k* takes the values k=5,10,20 in this paper. The motivation for this proposal comes from cases in which words with equivalent meaning, such as “running” and “jog”, exist in the text and there is a single activity class “run”. In order to take into account such fluctuation of words, the vector close to “run” $z_{run}$ is treated as the semantic vector of activity class “run”. In the process of combining multiple words, we apply the method to only the verb part. For instance, the class “Open door” has two words, “open” and “door”. In this case, we apply the method only to the part “open”, because we focus on the verbs in the proposal. We show the similar words for each activity class in each dataset in Appendix A.2. Words are assigned different colors depending on their relationship, red for synonyms and blue for antonyms, based on “Reverso Synonyms” (https://synonyms.reverso.net/synonym/).

## 4. Evaluation

In this section, we describe the experiments conducted to evaluate the performance of word embedding and expanded word embedding as semantic spaces for zero-shot activity recognition. The evaluation is based on two metrics:

- The recognition accuracy for unknown classes using word embedding and expanded word embedding as compared to the attribute vector, and
- The similarity between the word embedding semantic space/expanded word embedding semantic space and the attribute vector semantic space.

We first describe the datasets used for evaluation in Section 4.1, and then detail each evaluation method in Section 4.2.

### 4.1. Datasets

To evaluate the discussed methods, we use two public datasets and one proprietary dataset for activity recognition. These datasets are different in terms of types of activity classes and sensor position. A summary of these datasets is shown in Table 1 while more details are given in Section 4.1.1.

We first describe the datasets and the pre-processing for each one of them in Section 4.1.1, and we then describe the semantic vectors used in the evaluation in Section 4.1.2.

#### 4.1.1. Sensor Dataset and Pre-Processing

To evaluate the accuracy of estimating unknown classes, we use three datasets: the OPP dataset, the PAMAP2 dataset, and the HASC dataset (Table 1). For all three datasets, we first fill in zero-values for missing data, and then we normalize in the range [0,1] for each dimension. We use sliding windows to create feature vectors which is traditional method for extracting features in the activity recognition field. We now describe briefly each dataset.

**OPP dataset [32]** The Opportunity Activity Recognition Data Set (OPP) is a dataset that describes the behavior of people in the morning at work. The sensor data is collected from 4 subjects at a frequency of 30 Hz. This dataset is labeled with 4 different label types. We use the action label type “middle level activity class labels” which contains 17 activities. For the sliding time-window feature extraction, we use window time of 1 s and the slide time was set to 0.5 s by following what Hammerla Nils Y. et al. did [33]. The “Drill runs” data is not used. During feature extraction, variances and averages are extracted for each dimension. As a result of the pre-processing, we obtain 484 features in each feature vector.

**PAMAP2 dataset [34]** The Physical Activity Monitoring Data Set (PAMAP2) contains data from 9 subjects and 18 activities. The heart rate monitor sensor has a frequency of 9 Hz, while the data frequency of the other sensors is 100 Hz. The window time is set at 5.12 s and the slide time is set at 1 s for the sliding window feature extraction by following what Roggen Daniel did [34]. The “3D accelerators with scale of ±6 g” files and orientation readings have been deleted, as per the recommendations of the data provider. Extracting the variance and averages within each time window as a result of pre-processing, we obtain vectors of dimension 69.

**HASC dataset** The Human Activity Sensing Consortium (HASC) aims to construct a large-scale database of wearable sensors, and they run a challenge to collect acceleration sensor data from smartphones for 6 specific actions [35]. For this paper, we use the dataset collected in our laboratory according to the HASC challenge guidelines (http://hasc.jp/hc2010/HASC2010corpus/hasc2010corpus-en.html). Sensor readings are obtained from a 3-axis accelerometer at a frequency of 100 Hz. The dataset was collected by a single subject who, for each activity, recorded 5 sets each one lasting 20 s. The window time is set at 2 s and the slide time is set at 0.5 s for the sliding window feature extraction. Extracting the variance, averages, minimum and maximum within each time window as a result of pre-processing, we obtain vectors of dimension 12.

#### 4.1.2. Semantic Spaces

In this paper, we mainly evaluate and compare three kinds of semantic vector methods: attribute vector, word embedding, and expanded word embedding. In the following we describe the attribute vector and word embedding (which includes expanded embedding vector) used for the evaluation.

**Attribute vectors** For each of the evaluation datasets we define the attribute vectors shown in Table A1, Table A2 and Table A3 in Appendix A.1. These attribute vectors have been defined for the OPP and PAMAP2 dataset in [15] and we use the same attributes as the PAMAP2 dataset to create the attribute vector for HASC dataset which was not previously defined. Note this attribute value is treated as real value.

**Word embedding** For word embedding, we use the model wiki2vec (https://github.com/idio/wiki2vec), which is generated using English Wikipedia as corpus. This model provides 1000-dimensional vectors of all words created using word2vec. As mentioned before, the embedding word vectors for the activity classes in the datasets are extracted from this model. We use PCA to reduce the dimensions of the semantic vectors. As a result, it is 6 dimensions for HASCA dataset, 11 dimensions for OPP dataset and 18 dimensions for PAMAP2 dataset.

### 4.2. Method of Analysis and Evaluation Experiment

Having described the datasets and the semantic spaces used for the evaluation, we now explain how we evaluated the zero-shot learning method using word embedding for activity recognition. In this section, we explain the two evaluation contexts that were mentioned in the first part of Section 4.

#### 4.2.1. Evaluation of Unknown Class Estimation

To compare the performance of zero-shot Learning using the word embedding and the attribute vectors, we measure the accuracy of estimating unknown classes of each method. For word embedding, we prepare four methods: word embedding and extended word embedding with k=5,10 and 20. In all cases, we use SVRBM as projection method, as described in Section 3.2.

As an evaluation method, we use cross-validation. Usually, cross-validation for zero-shot learning evaluation considers different classes as unknown classes in each fold. We follow the procedure described in [15] for the OPP and PAMAP2 datasets, so we set 5 folds, each one with 3 or 4 unknown classes. For HASCA dataset, we set 2 unknown classes for each fold and prepare all possible pair combinations as unknown classes. This dataset has 6 classes in total, so we evaluate with 15 folds. The sets of unknown classes for each fold in each dataset are shown in Table 2, Table 3 and Table 4. The average number of instances of unknown and known classes in each fold is shown in Table 5. For the OPP dataset and PAMAP2 dataset. For HASCA dataset, the average number of instances is 1945. (2013 stay classes, 2014 walk classes, 1608 jog classes, 2010 skip classes, 2015 stair-up classes and 2012 stair-down classes.)

Finally, we use F-1 score as a metric to calculate the accuracy of the estimation. The formula for the metric is the following:

F-score=2×Precision×Recall/(Precision+Recall)

Precision=TP/(TP+FP),Recall=TP/(TP+FN)

In this formula, True Positive (TP) is the number of samples that belong to the target class and estimate the target class. False Positive (FP) is the number of samples that do not belong to the target class but estimate the target class. False Negative (FN) is the number of samples that do not belong to the target class and do not estimate it.

#### 4.2.2. Analysis of the Similarity of Semantic Spaces

The second evaluation objective is to compare the automatically generated word-embedding spaces to the hand-made attribute spaces. In this section, we explain how we calculate the similarity of these spaces.

As a first step, we calculate the *k*-most-similar classes for each class, for all semantic spaces. In this phase, we use a *k*-nearest neighbor algorithm. For the attribute vector space and the word embedding space, we calculate the top-3 nearest classes. For the expanded word embedding, we calculate the histogram of the nearest classes for each vector sample. Next, we calculate the degree of similarity between the word embedding space (with and without expanding) and the attribute space. The degree of similarity is calculated by examining the matching rate of nearest classes.

The reason for using this method is that the last step of the zero-shot method uses the nearest neighbor method in the semantic vector space, therefore the class similarity relationship is an important factor for the accuracy of the estimation.

## 5. Analysis and Experimental Results

### 5.1. Analysis of Projection Methods

Before evaluating the zero-shot learning method, we first evaluate the accuracy of the projection method in a setting without unknown classes. We use 30% of all data as test data. Table 6 shows the result of this non-zero-shot situation.

From the results in Table 6, we conclude that the zero-shot learning method performs well with the given datasets when there are no unknown classes. Moreover, the word embedding space has the best average performance among all three datasets.

### 5.2. Results of Unknown Class Estimation

**Figure 3:**
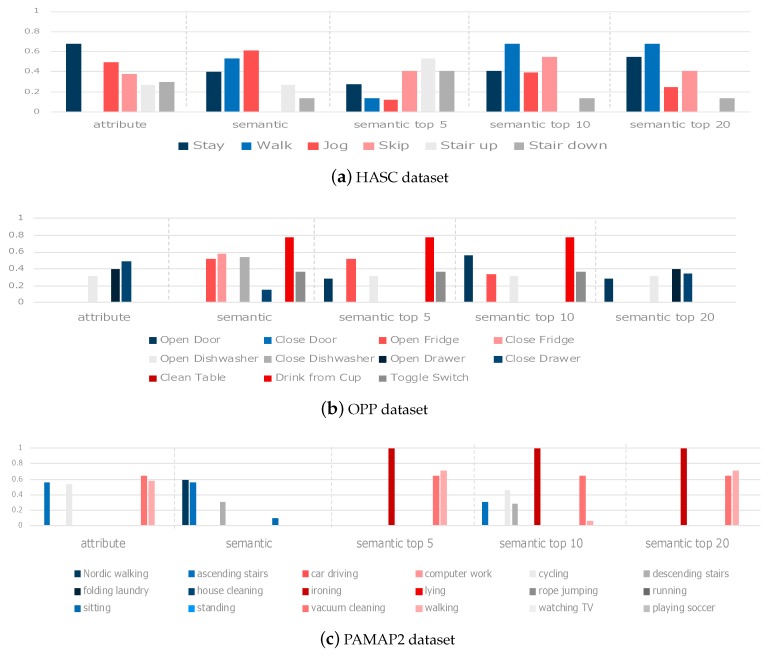
Average F-score of estimation for unknown activity classes.

After confirming that the projection method and the semantic spaces are suitable for activity classification, we evaluated the zero-shot learning scenario. As mentioned in Section 4.1.2, we use cross-validation and F1-Score for this evaluation. Figure 3 and Table 7 show the F1-Score when estimating unknown classes. Figure 3 shows the average F1-score for each class and Table 7 shows the overall average for each method. Boldface indicates the best score for each dataset.

From Table 7, we understand that using word embedding vectors gives the highest performance for all datasets. However, depending on the expanding parameter *k*, the performance can become lower than using attribute vector. Therefore, this parameter must be optimized when using expanded word embedding vector.

We now analyze the performance per class (Figure 3) focusing in the HASC dataset first. When the attribute vector is used, the “walk” class is not estimated. On the other hand, the performance of expanded word embedding for the classes “walk” and “stay” when k=5 is lower than using word embedding without expansion but it then improves gradually as *k* becomes larger. Conversely, the performance for the classes “Stair up” and “Stair down” is high initially, and then deteriorates as *k* becomes larger. Additionally, the classes “Clean Table”, “Drink From Cup” and “Toggle Switch” are not estimated by the attribute vector because their attributes are not shared with any known class Table A3. On the other hand, the method with embedding word vector estimates “Drink From Cup” and “Toggle Switch”.

Focusing on the OPP data result, we understand that the number of unknown classes that can be estimated differs between attribute and word embedding vectors. For attribute vectors, we can see that only 3 classes can be estimated, but for word embedding 6 classes can be estimated from the Figure 3b. However, we can see that this number decreases as we further expand the area of the embedding vector, and only 4 classes can be estimated when k=20. These four classes are similar to the estimated classes when using attribute vectors. Among all classes, the ”Drink form Cup” class has the highest F-score when using embedding vector until top10 expansion.

Finally, for the PAMAP2 data result, we understand that few classes are estimated by all methods. When we focus on estimated classes, “vacuum cleaning” and “walking” are estimated with good accuracy when using attribute vector and expanded embedding vector. In contrast to attribute vector and embedding word vector, the “ironing” class is only estimated when using expanded embedding vector.

Summarizing these results, we can achieve the highest average score when using embedding vector for all datasets. Also, we can see that the method with embedded word vector can estimate the classes which were impossible to be estimated by attribute vectors because of non-learnable attributes, which are not estimated. Focusing on class recognition accuracy, we observe that a different semantic vector performs better for different classes. In the Discussion (Section 6), we analyze what kind of word can be estimated and what is the impact of the choice of semantic vectors.

### 5.3. Correlation Analysis of Spatial Similarity

**Figure 4:**
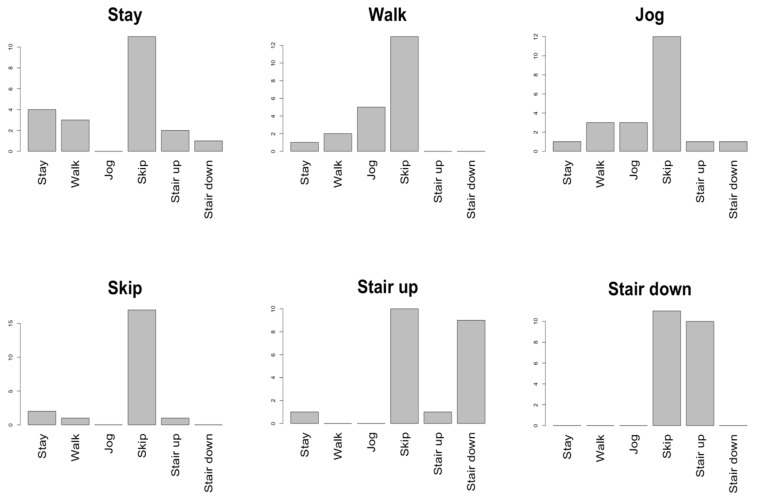
Histogram of the nearest classes appearing in HASC data for each sample in the set of expanded word embedding vectors.

**Figure A1:**
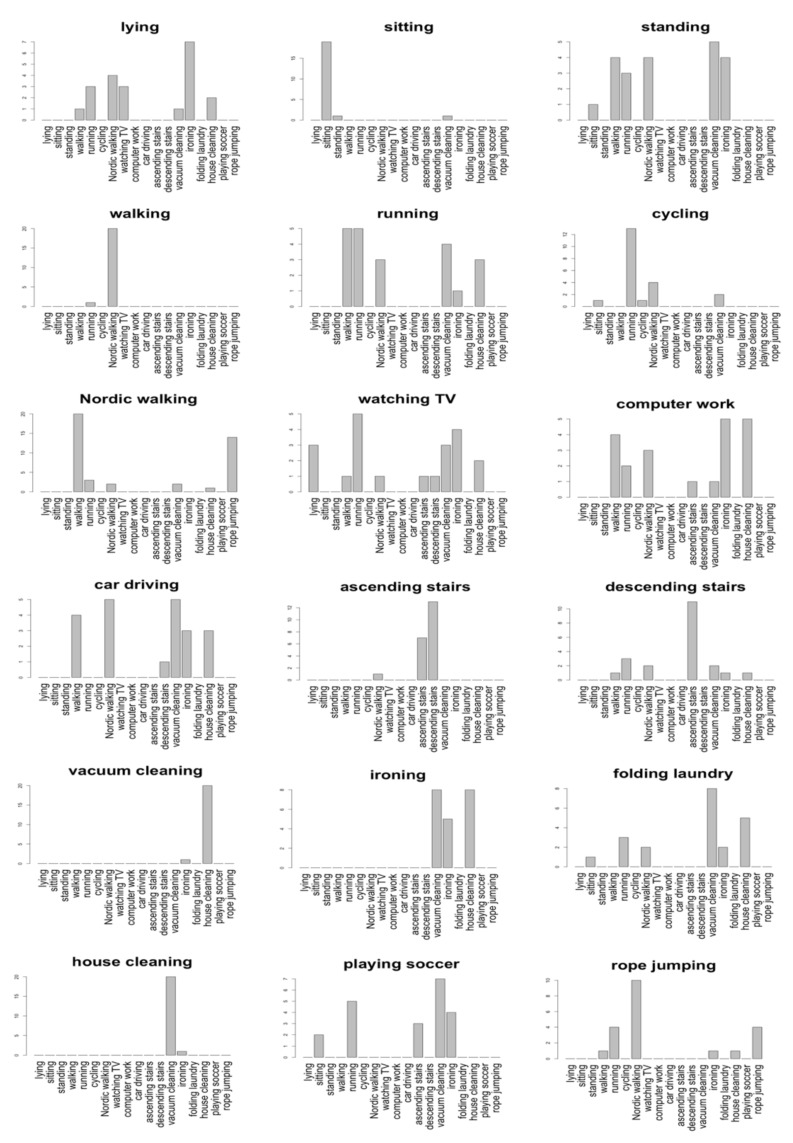
Result of the histogram of the nearest classes appearing in PAMAP2 data for each samples set for the word embedding area expansion.

**Figure A2:**
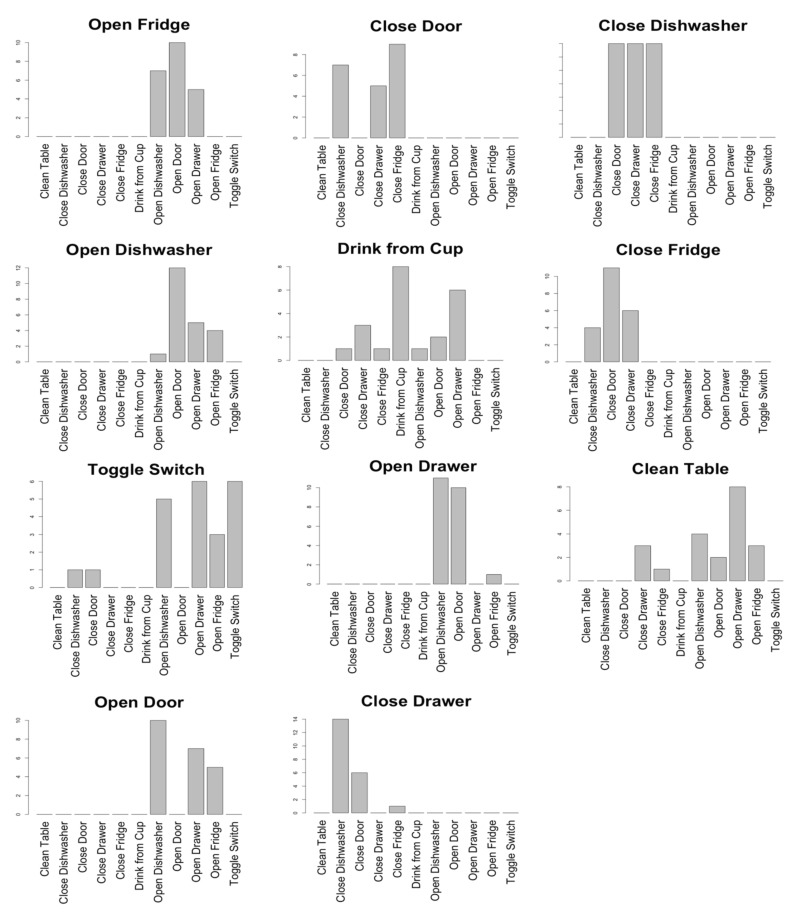
Result of the histogram of the nearest classes appearing in OPP data for each samples set for the word embedding area expansion.

To further understand the accuracy results obtained, we calculated the similarity between the attribute-space and the word embedding space with and without expanded as explained in Section 4.2.2. Table 8 shows the top-3 most similar classes when using the attribute vector space and the word embedding space for the HASC dataset, while Figure 4 shows the histogram of the most similar classes for the expanded word embedding spaces. The same results for the OPP dataset and the PAMAP2 dataset can be found in Table A7 and Figure A1, and Table A8 and Figure A2, respectively. To summarize these results, Table 9 shows the degree of similarity between each word embedding space and the attribute vector space.

Now we analyze the similarity among spaces for each dataset. We first focus on the *HASC dataset* (Table 8). When comparing the word embedding space to the attribute vector space, we can see that the classes “Stay”, “Walk”, “Stair up” and “Stair down” share 2 similar classes in both spaces. So we can consider them to be similar in both spaces. However, we observe that the classes “Jog” and “Skip” are not similar because they share only 1 similar class in both spaces. In the expanded word embedding space (Figure 4), all classes tend to be similar to the class “Skip”. Comparing among classes, in the word pairs “Stay” and “Walk”, “Stair up” and “Stair down”, we can see that the nearest classes are the same, so we can say that these classes are similar.

We now focus on the *PAMAP2 dataset* (Table A7 and Figure A1) to compare between the attribute vector space and word embedding space. Only the classes “vacuum cleaning” and “house cleaning” have two matching nearest words in both spaces, although for vacuum cleaning they match in order also. We can consider that these classes are similar between the attribute space and the word embedding space. Classes with only one matching nearest class are “descending stairs”, “ascending stairs”, “vacuum cleaning”, “ironing” and “cycling”. Focusing on Figure A1, the classes with the highest number of neighbors, are “vacuum cleaning” and “house cleaning”.

We now analyze the similarity between spaces in the *OPP dataset*. There are 11 action classes in total in the OPP dataset: Table A8 shows the result of comparing attribute vector space and word embedding. In this case, the attribute and the word embedding spaces differ greatly, as there are no matching words between spaces in this result. It is interesting to analyze this difference. In the OPP dataset, almost all classes are composed of a verb and an object. Focusing on each of these words, determining which are the nearest words depends on whether we focus on the verb or on the object. For instance, for the “Open Fridge” class, focusing on the verb “Open” gives nearest words in the attribute vector space and expanded word embedding space with the keyword Open, like Open Drawer and Open Door. However, in the word embedding space, the class nearest to the “Open Fridge” class is the one including the object “Fridge”. Focusing on the similarity between spaces reported on Table 9, we observe that the similarity between the word embedding space and the attribute space for this dataset is 0. Nevertheless, this is the case in which we obtain the highest accuracy (Table 7). From this result, we can see that there is no correlation between estimation accuracy and spatial similarity. Also, we can see why the automatically generated space can provide better accuracy than the hand-made attributes.

### 5.4. Summary of Results

We conclude with a summary of this section. This evaluation focused on analyzing the suitability of using word embedding vectors in Zero shot learning for sensor-based activity recognition. In three datasets featuring different types of activities, we found that using word embedding achieves equivalent or higher accuracy than the attribute vector. Moreover, we can see that the method with embedded word vector can estimate the classes which are impossible to be estimated by attribute because of non-shared attributes. However, the expanded embedding vector parameter *k* needs to be selected for each task.

When comparing the semantic spaces, we observed that the word embedding semantic space becomes more similar to the attribute vector space when we use expanded word embedding. From this result, it can be inferred that expanded word embedding brings the semantic space closer to the attribute vector space. However, in the OPP dataset, our results show we can get the best performance when using the word embedding without expanding. From this result, we observe the possibility to improve the accuracy of zero-shot sensor based activity recognition with word embedding.

## 6. Discussion

In this section we discuss the factors that influence the performance of word embedding vectors in zero-shot learning for sensor-based activity recognition. Particularly, we focus on 3 aspects: correctness of the meaning of embedded words, the impact of the choice of semantic vector and the effect of compound words.

### 6.1. Word Meaning in Word Embedding Vector

We discuss the relationship between the meaning of a word and performance based on the accuracy results reported in Section 5.2 and the analysis of the additional vectors included in the expanded word embedding space as reported in Appendix A.2. The relationship is based in the hypothesis that the performance improves when the expanded word embedding captures synonym words correctly. By analyzing the words of the top 5 expansion for the *HASC dataset*, we understand that embedding words are synonyms of the original activity word. However, the performance, compared to no expansion, decreases slightly. Interestingly, for the class “stay”, we find some vectors with opposite meaning (“go”, “leave”) in the expanding space but its score improves. Therefore, we conjecture that there is no correlation between meaning and performance. From the Figure 4 “Skip ” expanding word vector has the highest number of similar vectors for every class. From these results, the expanding word vectors might not work because the original words are close for each embedding vector.

For the *OPP dataset*, discussion is difficult because almost all activity classes are compound words. As mentioned before, the similarity of compund words can be given either by the verb or the object part. Suppose that we focus on the class “drink from cup”, we cannot see the relationship between the words easily. We will discuss the effect of compound words in Section 6.3.

For *PAMAP2 dataset*, the classes “cleaning” and “work” have 13 synonym words among the 20 words of the expanded semantic vector space. Although this is a lot for this dataset, we observe that the performance is not related to this since the classes “house cleaning” and “work” are not recognized. On the other hand, “vacuum cleaning”, “lying” and “walking” have better performance when expanding (Table 3) even if the latter two have fewer synonym words in the expansion. Therefore, we cannot find a correlation between meaning and performance for the PAMAP2 dataset neither.

In summary, we cannot observe any relation between correctness of the meaning of the semantic vector and the performance of the recognition.

### 6.2. Effect of the Choice of Semantic Space on the Performance

To discuss the relationship between the similarity of the word embedding semantic spaces with the attribute space and performance, we focus on Table 7 and Table 9. We understand that the performance on the OPP dataset declines by expanding, but for PAMAP2 and HASCA dataset we can see the opposite. However, for HASCA dataset the similarity with the attribute space was reduced with expanding whereas for the OPP and PAMAP2 datasets the similarity increased with expanding. However, the similarity with the attribute space seems to be not related with a better performance. In fact, this is observed with the OPPortunity Dataset. The word embedding space without expansion has 0 similarity with the attribute space, yet it has the best performance among all spaces. As the space becomes more similar to the attribute space, the performance decreases until it reaches a similar performance as the attribute space. This result suggests that the word embedding may be able to capture better characteristics about each activity than it was possible to describe with the attributes.

In contrast, in PAMAP2 dataset, becoming more similar to the attribute space implies a better performance. In fact, we can see the similarity between “walking” and “house cleaning” in the embedding space is the same as in the attribute space (Table A1). We observe that the attributes designed for PAMAP2, are detailed and include specific body postures and limbs movements combined with objects, whereas the attributes of the OPP dataset are just decomposition of the activity words. These results suggests that the word embedding may be able to capture some of these details.

### 6.3. Compound Words

We have seen that in the OPP dataset, the word embedding has a better performance than the attribute space and that, as the word embedding space becomes more similar to that of the attribute space, the performance is reduced. We now analyze this result in the light of the nature of the action words in this dataset which, as we have mentioned before, are compound words.

Before we examine each part separately, let us review the activity classes of the OPP dataset. These classes are all in the form “verb” + “object”, and many activity classes differ only in combination. For example, “open door” and “close door” are classes that share objects, while “open door” and “open fridge” are classes sharing their verbs.

In the word embedding method, we usually focus on the “object” part when classifying an activity. However, applying the idea of expanded word embedding gives rise to many patterns in which area vectors focus on the “verb” parts. This is also the focus of the attribute space, as was seen in Table A8.

**Figure 5:**
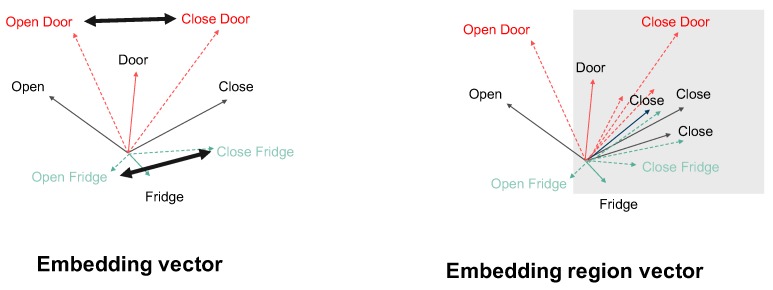
Difference between embedding vector and embedding region vector.

To visualize this, see Figure 5. As mentioned in Section 4.1.2, for labels composed of two or more words, the semantic vector takes the average of the corresponding word vectors. For this reason, in the word embedding space, the word vector for the class “Open Door” is closer to the word vector of the class sharing the object part, “Close Door”, than to the words sharing the verb part like “Open Fridge” (left part of Figure 5). However, applying the expanded word embedding technique reduces the distance between the classes “Open Door” and “Open Fridge” (right part of Figure 5).

Therefore, when we search in the neighborhood of a vector in the word embedding space, its nearest neighbors tend to focus on the “object” part of the class, while in the space of word embedding area expansion, the focus is around the verb part (Table A8 and Figure A2). We observe that in the sensor vector space, the distance between classes using the same object is closer than that of the classes with the same motion. For instance the movement sensor readings and object sensor measurements of “open Door” and “close Door” are more similar than those of “open Door” and “open Dishwasher”. Therefore, for sensor activity recognition, the embedding vector is more efficient and it is more sensible to apply it as semantic vector.

Based on this analysis, we discuss now the impact of knowledge on the accuracy of zero-shot activity recognition. As we have observed, in the OPP dataset, the word embedding space offers better performance than both the attribute space and the expanded word embedding space. In other words, using a semantic vector that focuses on the “object” part leads to better performance. This is owed to the fact that the OPP dataset includes object sensor information in the feature vector, which is very important for class identification. This information is translated to the semantic vector space. We conclude therefore that when similar information that is crucial for class identification exists both in the feature and semantic spaces, then the impact on performance is positive. This knowledge is valuable for future research in zero-shot learning for sensor activity recognition. Consider as an example the fact that, prior to gathering data, we must determine in advance the activity classes and the sensor types of the dataset. Apart from considering the requirements best suited for the application system at hand, with this knowledge we can also a priori aim for better performance.

### 6.4. Future Work

It is also possible that the test set contains instances from both known and unknown classes. In this paper, we did not evaluate this situation but this situation is important for applications [5]. There are researches in this situation, so we will evaluate this situation as the future work.

Although the performance of unknown class prediction is not as high as expected, predicting complex activity, even in a traditional supervised setting, is a difficult task, like house activities and working activities. There are many factors that difficult this recognition, for example, different place layout, different objects, or different people even if we try to predict the same activities. We think that the technology of activity recognition needs zero-shot learning approach which estimates attribute classes or semantic meaning to get general knowledge about the activities. Also collecting training data is too tough and difficult process for application, especially for complex activities, so it is important to consider how to make this process efficient.

We can argue that the expanding word vector is the same as the embedding vector. This is because the expanding word embedding vectors are selected around the embedding vector, so their values are very similar and the closest vector would have been from the same class if no expansion had been done. However, our assumption is that the feature vectors have similar motions in multiple classes difficulting the projection task. This is why we need to make semantic vector general by expanding word embedding to help the projection task from feature vector into semantic vector.

## 7. Conclusions

In this paper, we compared three semantic representations for zero-shot sensor-based activity recognition: attribute vector, embedding vector and expanded word embedding vector. To solve complications that arise due to the use of word embedding as semantic vector, we introduced the expanded word embedding. We evaluated word embeddings with three datasets by performing: (a) a comparison of the accuracy of the estimation of unknown classes between the attribute vector method and the word embedding methods; and (b) an analysis of the similarity between the semantic spaces obtained with each method.

Our results demonstrate that using word embedding vector with expanded word embedding has advantages over attribute vectors. First, we achieved the highest average score when using embedding vector for all datasets. Also, the method with embedded word vector can estimate the classes which are impossible to be estimated by with the attribute vector since some of their attributes are not represented in the training classes. For zero-shot learning, the correlation between the space of sensor feature vector and the space of semantic vector has a big impact on performance. As an advantage, this correlation is larger when by using embedding word vector than when using attribute vector. These results indicate that, compared to the hand-generated attribute vector, the use of word embedding is potentially more efficient, as it is generated automatically.

Also we obtained some useful knowledge for activity recognition. The set space of expanded word embedding, compared to the set space of expanded word embedding, is closer to the attribute set space. This result means expanding brings the semantic space closer to human knowledge. However, in this result, we see that the attribute vector does not always improve the accuracy of Zero-shot learning. This result is the answer of the first and second questions raised in the introduction section.

With respect to the performance of the semantic spaces, being similar to human-made attributes should not be the goal when generating the embedding vector. These results are essential for selecting which activity classes and sensor data to include in learning and testing datasets when applying the zero-shot method for activity recognition.

Due to the efficiency of the method that we discussed in this paper, the direction of our future research will align with the promising zero-shot learning for activity recognition based on word embedding generated from text data.

## Figures and Tables

**Table 1:** Activity classes and sensor types of each dataset1-4.

Dataset	Activity Class Type	Sensor Type
HASC	Basic (Walk, Run, Jog, Skip, Stair up and Stair down)	Accelerometer worn on arm (including acceleration sensor) on arm
OPP	Middle (open door, close door, open fridge, close fridge, open dishwasher, close dishwasher, open drawer, close drawer, clean table, drink from cup and toggle switch)	Ambient sensors and wearable sensors (including IMUs and acceleration sensor) on 7 points
PAMAP2	Living (watching TV, house cleaning, lying, sitting, standing, walking, running, cycling, Nordic walking, computer work, car driving, ascending stairs, descending stairs, vacuum cleaning, ironing, folding laundry, playing soccer and rope jumping)	Wearable sensors (including IMUs and heart rate monitor sensor) on 3 points

**Table 2:** Foldings of the OPP classes.

Fold	Unknown Classes
fold 1	Close Drawer, Clean Table, Toggle Switch
fold 2	Open Fridge, Open Door, Close Drawer
fold 3	Drink from Cup, Open Drawer, Close Dishwasher
fold 4	Close Drawer, Close Door, Open Door
fold 5	Close Fridge, Open Dishwasher, Close Door

**Table 3:** Foldings of the PAMAP2 classes.

Fold	Unknown Classes
fold 1	watching TV, house cleaning, standing, ascending stairs
fold 2	walking, rope jumping, sitting, descending stairs
fold 3	playing soccer, lying, vacuum cleaning, computer work
fold 4	cycling, running, Nordic walking
fold 5	ironing, car driving, folding laundry

**Table 4:** Foldings of the HASC classes. The values in the parenthesis is number of instances of each class.

Fold	Unknown Classes	Fold	Unknown Classes
fold 1	Stay (2013), Walk (2014)	fold 9	Jog, Stair up
fold 2	Stay, Jog (1608)	fold 10	Skip, Stair up
fold 3	Walk, Jog	fold 11	Stay, Stair down (2012)
fold 4	Stay, Skip (2010)	fold 12	Walk, Stair down
fold 5	Walk, Skip	fold 13	Jog, Stair down
fold 6	Jog, Skip	fold 14	Skip, Stair down
fold 7	Stay, Stair up (2015)	fold 14	Skip, Stair down
fold 8	Walk, Stair up	fold 15	Stair up, Stair down

**Table 5:** Statistics on the number of instances and classes that belong to seen and unseen classes in each fold in the three datasets.

Fold	Number of Instances				Number of Classes			
	**OPP**		**PAMAP2**		**OPP**		**PAMAP2**	
**Classes**	**Seen**	**Unseen**	**Seen**	**Unseen**	**Seen**	**Unseen**	**Seen**	**Unseen**
fold 1	5868	1338	16377	3073	8	3	14	4
fold 2	4986	2220	13661	5789	8	3	14	4
fold 3	4647	2559	15770	3680	8	3	14	4
fold 4	5087	2119	14927	4523	8	3	15	3
fold 5	5525	1681	17065	2385	8	3	15	3

**Table 6:** Evaluation of the methods without unknown classes. F1-Score when using 30% as test data.

	HASC	OPP	PAMAP2
supervised_SVM	0.84	0.85	0.92
ZSL_attribute	0.91	0.78	0.91
ZSL_embedding	0.92	0.92	0.91

**Table 7:** Average F-score of the estimation of activity classes with 3 datasets. For each dataset we show the 5 methods using different semantic spaces.

	HASC	OPP	PAMAP2
attribute	0.35	0.11	0.13
semantic	0.32	**0.27**	0.09
semantic_top5	0.31	0.21	0.13
semantic_top10	**0.36**	0.21	**0.15**
semantic_top20	0.33	0.12	0.13

**Table 8:** Top 3 nearest words in the attribute space (left) and the embedding space (right) for the HASC dataset.

		1st	2nd	3rd	1st	2nd	3rd
1	Stay	Walk	Jog	Stair down	Walk	Jog	Stair up
2	Walk	Jog	Stair down	Stair up	Jog	Stay	Stair down
3	Jog	Skip	Walk	Stair down	Walk	Stay	Stair up
4	Skip	Jog	Stair up	Stair down	Walk	Jog	Stay
5	Stair up	Stair down	Skip	Jog	Stair down	Jog	Stay
6	Stair down	Stair up	Skip	Jog	Stair up	Jog	Walk

**Table 9:** Degree of similarity between the embedding space and the attribute vector space, i.e., the ratio of matching words in the nearest classes for each class in Figure 4, Figure A1 and Figure A2.

	HASC	OPP	PAMAP2
word embedding	4/6	0/11	0/11 5/18
expanded word embedding	1/6	2/11	8/18

## Appendix A

### Appendix A.1. Attribute Vectors

In this section, we show 3 attribute vectors for each dataset.

**Table A1:** Attribute vectors of the HASC dataset.

	Act	Motion	Static	Cyclic Motion	Intense Motion	Translation Motion	Body Up	Body Down	Body in Place	Arms Motion	Arms Static	Arms Bent	Arms Straight	Arm Bent Straight Transform	Legs Motion	Legs Static	Legs Bent	Legs Straight	Legs Bent Straight Transform	Legs Alternate Move Forward	Legs Move Up and/or Down	Stairs
1	Stay	0	1	0	0	0	0	0	1	0	1	0	1	0	0	1	0	1	0	0	0	0
2	Walk	1	0	1	0	1	0	0	0	1	0	0	1	1	1	0	1	0	1	1	0	0
3	Jog	1	0	1	1	1	0	0	0	1	0	1	0	1	1	0	1	0	1	1	0	0
4	Skip	1	0	1	1	1	1	1	0	1	0	1	0	1	1	0	1	0	1	1	1	0
5	Stair up	1	0	1	0	0	1	0	0	1	0	1	0	1	1	0	1	0	1	1	1	1
6	Stair down	1	0	1	0	0	0	1	0	1	0	1	0	1	1	0	1	0	1	1	1	1

**Table A2:** Attribute vectors of the PAMAP dataset from [15].

	Act	Motion	Static	Cyclic Motion	Intense Motion	Translation Motion	Free Motion	Body Vertical	Body Incline	Body Horizontal	Body Forward	Body Backward	Body Up	Body Down	Body in Place	Torso Transform	Arms Motion	Arms Static	Arms Bent	Arms Stranght	Arms Bent Straight Ransform	Hands Hold Something	Legs Motion	Legs Static	Legs Bent	Legs Straight	Legs Bent Straight Transform	Legs Alternate Move Forward	Legs Move Up and/or Down	Seat	Bike	Poles	Television	Computer	Car	Stairs	Vacuum	Iron	Clothes	Soccer	Rope	Indoor	Outdoor
1	lying	0	1	0	0	0	0	0	0	1	0	0	0	0	1	0	1	1	1	1	1	0	1	1	1	1	1	0	0	0	0	0	0	0	0	0	0	0	0	0	0	1	1
2	sitting	0	1	0	0	0	0	1	0	0	0	0	0	0	1	0	1	1	1	1	1	0	0	1	1	0	0	0	0	1	0	0	0	0	0	0	0	0	0	0	0	1	1
3	standing	0	1	0	0	0	0	1	0	0	0	0	0	0	1	0	1	1	1	1	1	0	0	1	0	1	0	0	0	0	0	0	0	0	0	0	0	0	0	0	0	1	1
4	walking	1	0	1	0	1	0	1	0	0	1	0	0	0	0	0	1	0	0	0	1	0	1	0	0	0	1	1	0	0	0	0	0	0	0	0	0	0	0	0	0	1	1
5	running	1	0	1	1	1	0	1	0	0	1	0	0	0	0	0	1	0	1	0	0	0	1	0	0	0	1	1	0	0	0	0	0	0	0	0	0	0	0	0	0	0	1
6	cycling	1	0	1	0	1	0	0	1	0	1	0	0	0	0	0	0	1	1	1	0	1	1	0	0	0	1	0	0	1	1	0	0	0	0	0	0	0	0	0	0	0	1
7	Nordic walking	1	0	1	0	1	0	1	0	0	1	0	0	0	0	0	1	0	0	0	1	1	1	0	0	0	1	1	0	0	0	1	0	0	0	0	0	0	0	0	0	0	1
8	watching TV	0	1	0	0	0	0	1	1	0	0	0	0	0	1	0	1	1	1	1	1	0	1	1	1	1	1	0	0	1	0	0	1	0	0	0	0	0	0	0	0	1	0
9	computer work	0	1	0	0	0	0	1	1	0	0	0	0	0	1	0	1	1	1	1	1	1	1	1	1	0	0	0	0	1	0	0	0	1	0	0	0	0	0	0	0	1	0
10	car driving	0	1	0	0	0	0	1	0	0	1	0	0	0	0	0	1	0	1	0	0	1	1	1	1	0	0	0	0	1	0	0	0	0	1	0	0	0	0	0	0	0	1
11	ascending stairs	1	0	1	0	0	0	1	1	0	1	0	1	0	0	0	1	0	1	1	1	1	1	0	0	0	1	1	1	0	0	0	0	0	0	1	0	0	0	0	0	1	1
12	descending stairs	1	0	1	0	0	0	1	1	0	1	0	0	1	0	0	1	0	1	1	1	1	1	0	0	0	1	1	1	0	0	0	0	0	0	1	0	0	0	0	0	1	1
13	vacuum cleaning	1	0	0	0	0	1	1	1	0	1	1	0	0	0	1	1	0	1	1	1	1	1	0	1	1	1	0	0	0	0	0	0	0	0	0	1	0	0	0	0	1	0
14	ironing	1	0	0	0	0	1	1	1	0	0	0	0	0	1	1	1	0	1	1	1	1	1	0	1	1	0	0	0	1	0	0	0	0	0	0	0	1	1	0	0	1	0
15	folding laundry	1	0	0	0	0	1	1	1	0	0	0	0	0	1	1	1	0	1	1	1	1	1	1	1	1	1	0	0	1	0	0	0	0	0	0	0	0	1	0	0	1	0
16	house cleaning	1	0	0	0	0	1	1	1	0	1	1	1	1	1	1	1	0	1	1	1	1	1	0	1	1	1	0	1	1	0	0	0	0	0	0	1	0	1	0	0	1	0
17	playing soccer	1	0	0	1	0	1	1	1	0	1	0	1	1	0	1	1	0	1	1	1	0	1	0	1	1	1	1	0	0	0	0	0	0	0	0	0	0	0	1	0	0	1
18	rope jumping	1	0	0	1	0	0	1	0	0	0	0	1	1	1	0	1	0	1	1	1	1	1	0	1	1	1	0	1	0	0	0	0	0	0	0	0	0	0	0	1	0	1

**Table A3:** Attribute vectors of the OPP dataset from [15].

	Act	Open	Close	Clean	Drink	Toggle	Door	Fridge	Dishwasher	Drawer	Table	Cup	Switch
1	Open Door	1	0	0	0	0	1	0	0	0	0	0	0
2	Close Door	0	1	0	0	0	1	0	0	0	0	0	0
3	Open Fridge	1	0	0	0	0	0	1	0	0	0	0	0
4	Close Fridge	0	1	0	0	0	0	1	0	0	0	0	0
5	Open Dishwasher	1	0	0	0	0	0	0	1	0	0	0	0
6	Close Dishwasher	0	1	0	0	0	0	0	1	0	0	0	0
7	Open Drawer	1	0	0	0	0	0	0	0	1	0	0	0
8	Close Drawer	0	1	0	0	0	0	0	0	1	0	0	0
9	Clean Table	0	0	1	0	0	0	0	0	0	1	0	0
10	Drink from Cup	0	0	0	1	0	0	0	0	0	0	1	0
11	Toggle Switch	0	0	0	0	1	0	0	0	0	0	0	1

### Appendix A.2. Similarity Words for Each Activity Classes

**Table A4:** Top 20 most similar words for each activity class in the HASC dataset. The symbol ** indicates that “DBPEDIA_ID/” has been omitted. The color of red represent synonyms and blue for antonyms.

Activity Class	Top 5	Top 10	Top 20
down	back, down”, down), down;, off	away, cut, down?, shut, upside	backwards, dragged, falling, finally, off;, out, slid, slowly, up, up”
jog	jogged, jogging, jogs, stroll, walk	bends, detour, half-mile, north-northwesterly, walking	driveway, eastbound, intersecting, intersects, lope, nap, ramp, straightens, swerves, veers
skip	go, miss, skipped, skipping, skips	Brier, curling, get, ignore, repeat	**the_Brier, Parsoid, Scribunto, append, editpreview, manually, redo, start, try, unwatch
stay	go, leave, remain, staying, stays	continue, settle, spend, wait, stayed	agrees, come, decide, decides, get, kept, marry, move, sit, vacation
up	back, up”, up:, up;, up?	down, forth, off, start, up)	0:), aside, carrots→, finally, just, out, quickly, start, them, up!
walk	path, stroll, walked, walking, walks	**20_kilometres_walk, go, jump, trek, wander	barefoot, climb, distance, kilometres, pull, relax, sit, strolling, swim, throw

**Table A5:** Top 20 most similar words for each activity class in the OPP dataset. The symbol ** indicates that “DBPEDIA_ID/” has been omitted. The color of red represent synonyms and blue for antonyms.

Activity Class	Top 5	Top 10	Top 20
clean	cleaning, cleans, messes, tidy, wash	fixing, remove, rinse, scrubbing, washing	bring, clean-up, cleanup, fix, lighten, mess, recycle, soak, tidying, trash
close	closer, closest, closure, proximity, strong	busy, closes, contact, relative, ties	another, clear, closing, connection, friend, friends, keep, move, nearer, reopen
toggle	buttons, on/off, switch, toggled, toggles	disable, double-click, knob, right-click, show/hide	“Cite”, Rightclick, button, button;, doubleclicking, joystick, right-clicking, scrollbars, tabs, touchpad
drink	beer, beverage, beverages, drinks, nonalcoholic	carbonated, drank, soda, sodas, vodka	**soft_drink, **soft_drinks, caffeinated, coffee, cola, fizzy, juice, juices, lemonade, non-carbonated
open	**open_source, access, enclosed, space, spaces	“open, allow, doors, **open_plan, **open_source_software	Access, **Open-source_software, **open_content, accessible, create, door, extension, internal, opening, repository

**Table A6:** Top 20 similar words for each activity class in the PAMAP2 dataset. The symbol ** indicates that “DBPEDIA_ID/” has been omitted; * indicates that “_New_York_State_Legislature” has been omitted. The color of red represent synonyms and blue for antonyms.

Activity Class	Top 5	Top 10	Top 20
ascending	ascend, ascends, descending, dhaivatam, panchamam	madhyamam, parallage, pentatonic, phthongos, scale)	(ascending, **chromatic_scale, **swara, arching, kaisiki, nishadham, rishabham, sadharana, shuddha, tetrachord
cleaning	clean, cleaners, cleans, laundry, washing	drying, polishing, repairing, scrubbing, vacuuming	Cleaning, cleanup, cleaner, dishwashers, ironing, plumbing, rinse, tidy, tidying, wash
cycling	Cycling, **2013_in_women’s_road_cycling, **2014_in_women’s_road_cycling, **Road_bicycle_racing, **track_cycling	**2013_UCI_Road_World_ Championships_Women’s_road_race, **List_of_women’s_road_bicycle_ races, **road_cycling , UCI, bicycle	BMX, **BMX_racing, **Mountain_bike_racing, **Track_cycling, **cyclocross, **race_stage, **road_bicycle_racing,**stage_race, cyclocross, cyclocross
descending	ascend, ascending, ascends, descend, downwards	climbs, descends, parallage, phthongos, scale)	(ascending, climb, descents, dhaivatam, downward, panchamam, steep, steeper, tetrachord, upward
driving	car, car’s, driver, drove, speeding	cars, drive, driver’s, drivers, stickshift	braking, burnouts, cornering, driver’s, drives, driving), motor, parked, vehicle, vehicle’s
folding	Folding, fold, folded, removable, sliding	adjustable, backrest, foldable, hinged, onepiece	Dbox, **protein_folding, armrests, chamferboards, folds, footrests, helical, laminated, nonslip, rearwards,
ironing	laundry, pillows, sewing, towels, washing	cleaning, dryer, mattresses, utensils, wash	**clothes_dryer, bathroom, clothes, cloths, dishwashers, dryers, napkins, towel, vacuuming, washable
jumping	**show_jumping, Jumping, dressage, ski, skiing	**eventing, **ski_jumping, Ski, downhill, eventing	**Show_jumping, **Ski_jumping, **dressage, **ski_cross, **ski_jumping_hill, jumper, leaping, paraNordic, snowboard, snowboarding
lying	bed, crying, dragged, lie, sleeping	beside, bluff, hiding, lied, scared	asleep, cheating, dragging, dumped, hugging, knees, pillow, screaming, slept, telling
playing	Playing, performing, play, played, plays	**full_back_(association_football), footballing, player, semiprofessional, singing	**Full_back_(association_football), **forward_(football), **midfield, career, club, midfield, playes, professionally, semiprofessionally, touring
running	cross, extending, ran, run, runs	Running, line, operating, stretch, walking	**road_running, **track_running, connecting, parallel, pulling, stretched, stretching, switching, walk, winding
sitting	**168th*, **169th*, **171st*, **172nd*, **176th*	**166th*, **167th*, **170th*, **173rd*, **174th*	165th, 167th, 178th, **164th*, **165th*, **175th*, **177th*, **178th*, **179th*, **180th*
standing	holding, kneeling, seated, stands, stood	crouching, dressed, modified, sitting, stand	facing, hands, hangs, hung, ovation, resting, sits, smiling, unless, wearing
walking	jogging, trail, trails, walk, walks	accessible, bicycling, biking, hiking, strolling	**walking, barefoot, bike, distance, horseback, picnicking, rollerblading, sidewalk, stroll, walked
watching	enjoy, enjoying, laughing, watch, watched	chatting, crazy, listening, seeing, viewing	crying, enjoys, fun, kid, kids, loved, noticing, scared, screaming, staring
work	work:, work;, working, works, work	endeavors, job, necessary, studies, work)	Work, creations, efforts, endeavor, endeavours, experience, expertise, focus, oeuvre, research

### Appendix A.3. Spatial Similarity

**Table A7:** Top 3 nearest words in the attribute space (left) and the embedding space (right) for the PAMAP2 dataset.

		1st	2nd	3rd	1st	2nd	3rd
1	lying	standing	sitting	watching TV	descending stairs	car driving	ascending stairs
2	sitting	standing	computer work	watching TV	descending stairs	ascending stairs	standing
3	standing	sitting	lying	watching TV	descending stairs	ascending stairs	sitting
4	walking	Nordic walking	running	descending stairs	descending stairs	watching TV	playing soccer
5	running	walking	Nordic walking	cycling	standing	descending stairs	sitting
6	cycling	running	Nordic walking	walking	running	standing	vacuum cleaning
7	Nordic walking	walking	running	descending stairs	watching TV	descending stairs	walking
8	watching TV	computer work	sitting	lying	car driving	sitting	computer work
9	computer work	watching TV	sitting	standing	playing soccer	descending stairs	car driving
10	car driving	sitting	computer work	standing	descending stairs	ascending stairs	lying
11	ascending stairs	descending stairs	walking	Nordic walking	descending stairs	folding laundry	sitting
12	descending stairs	ascending stairs	walking	Nordic walking	ascending stairs	car driving	sitting
13	vacuum cleaning	house cleaning	folding laundry	ironing	house cleaning	folding laundry	sitting
14	ironing	folding laundry	vacuum cleaning	house cleaning	folding laundry	ascending stairs	standing
15	folding laundry	ironing	vacuum cleaning	house cleaning	ascending stairs	ironing	descending stairs
16	house cleaning	vacuum cleaning	folding laundry	ironing	vacuum cleaning	sitting	folding laundry
17	playing soccer	rope jumping	vacuum cleaning	house cleaning	descending stairs	computer work	ascending stairs
18	rope jumping	playing soccer	house cleaning	ascending stairs	standing	sitting	descending stairs

**Table A8:** Top 3 nearest words in the attribute space (left) and the embedding space (right) for the OPP dataset.

		1st	2nd	3rd	1st	2nd	3rd
1	Open Door	Open Drawer	Open Dishwasher	Open Fridge	Close Drawer	Close Door	Open Fridge
2	Close Door	Close Drawer	Close Dishwasher	Close Fridge	Open Door	Clean Table	Drink from Cup
3	Open Fridge	Open Drawer	Open Dishwasher	Open Door	Close Fridge	Open Door	Open Drawer
4	Close Fridge	Close Drawer	Close Dishwasher	Close Door	Open Fridge	Close Drawer	Open Door
5	Open Dishwasher	Open Drawer	Open Fridge	Open Door	Close Dishwasher	Open Drawer	Toggle Switch
6	Close Dishwasher	Close Drawer	Close Fridge	Close Door	Open Dishwasher	Close Drawer	Close Fridge
7	Open Drawer	Open Dishwasher	Open Fridge	Open Door	Close Drawer	Open Dishwasher	Open Fridge
8	Close Drawer	Close Dishwasher	Close Fridge	Close Door	Open Drawer	Open Door	Close Dishwasher
9	Clean Table	Toggle Switch	Drink from Cup	Close Drawer	Close Door	Drink from Cup	Open Fridge
10	Drink from Cup	Toggle Switch	Clean Table	Close Drawer	Close Door	Open Fridge	Open Door
11	Toggle Switch	Drink from Cup	Clean Table	Close Drawer	Open Dishwasher	Close Dishwasher	Drink from Cup

## References

[1] Lara O.D., Labrador M.A. (2012). A survey on human activity recognition using wearable sensors. IEEE Commun. Surv. Tutor..

[2] Ransing R.S., Rajput M. Smart home for elderly care, based on Wireless Sensor Network. Proceedings of the 2015 International Conference on Nascent Technologies in the Engineering Field (ICNTE).

[3] Aehnelt M., Wegner K. Learn but work!: Towards self-directed learning at mobile assembly workplaces. Proceedings of the 15th International Conference on Knowledge Technologies and Data-driven Business.

[4] Perez A.J., Labrador M.A., Barbeau S.J. (2010). G-sense: A scalable architecture for global sensing and monitoring. IEEE Netw..

[5] Socher R., Ganjoo M., Manning C.D., Ng A. (2013). Zero-shot learning through cross-modal transfer. Advances in Neural Information Processing Systems.

[6] Zhang Z., Saligrama V. (2016). Zero-shot recognition via structured prediction. European Conference on Computer Vision.

[7] Fu Y., Yang Y., Hospedales T., Xiang T., Gong S. (2015). Transductive multi-label zero-shot learning. arXiv.

[8] Norouzi M., Mikolov T., Bengio S., Singer Y., Shlens J., Frome A., Corrado G.S., Dean J. (2013). Zero-shot learning by convex combination of semantic embeddings. arXiv.

[9] Akata Z., Reed S., Walter D., Lee H., Schiele B. Evaluation of output embeddings for fine-grained image classification. Proceedings of the IEEE Conference on Computer Vision and Pattern Recognition.

[10] Bucher M., Herbin S., Jurie F. (2016). Improving semantic embedding consistency by metric learning for zero-shot classiffication. European Conference on Computer Vision.

[11] Lampert C.H., Nickisch H., Harmeling S. Learning to detect unseen object classes by between-class attribute transfer. Proceedings of the 2009 IEEE Conference on Computer Vision and Pattern Recognition.

[12] Guadarrama S., Krishnamoorthy N., Malkarnenkar G., Venugopalan S., Mooney R., Darrell T., Saenko K. Youtube2text: Recognizing and describing arbitrary activities using semantic hierarchies and zero-shot recognition. Proceedings of the IEEE International Conference on Computer Vision.

[13] Alexiou I., Xiang T., Gong S. Exploring synonyms as context in zero-shot action recognition. Proceedings of the 2016 IEEE International Conference on Image Processing (ICIP).

[14] Xu X., Hospedales T., Gong S. Semantic embedding space for zero-shot action recognition. Proceedings of the 2015 IEEE International Conference on Image Processing (ICIP).

[15] Xian Y., Akata Z., Sharma G., Nguyen Q., Hein M., Schiele B. Latent embeddings for zero-shot classification. Proceedings of the IEEE Conference on Computer Vision and Pattern Recognition.

[16] Foerster F., Smeja M., Fahrenberg J. (1999). Detection of posture and motion by accelerometry: A validation study in ambulatory monitoring. Comput. Hum. Behav..

[17] Vishwakarma S., Anupam A. (2013). A survey on activity recognition and behavior understanding in video surveillance. Vis. Comput..

[18] Tapia E.M., Intille S.S., Haskell W., Larson K., Wright J., King A., Friedman R. Real-time recognition of physical activities and their intensities using wireless accelerometers and a heart rate monitor. Proceedings of the 2007 11th IEEE International Symposium on Wearable Computers.

[19] He Z., Jin L. Activity recognition from acceleration data based on discrete consine transform and svm. Proceedings of the 2009 IEEE International Conference on Systems, Man and Cybernetics.

[20] Parkka J., Ermes M., Korpipaa P., Mantyjarvi J., Peltola J., Korhonen I. (2006). Activity classification using realistic data from wearable sensors. IEEE Trans. Inf. Technol. Biomed..

[21] Peng L., Chen L., Ye Z., Zhang Y. (2018). Aroma: A deep multi-task learning based simple and complex human activity recognition method using wearable sensors. Proc. ACM Interact. Mob. Wearable Ubiquitous Technol..

[22] Wang J., Chen Y., Hao S., Peng X., Hu L. (2019). Deep learning for sensor-based activity recognition: A survey. Pattern Recognit. Lett..

[23] Kwon Y., Kang K., Bae C. (2014). Unsupervised learning for human activity recognition using smartphone sensors. Expert Syst. Appl..

[24] Cook D., Feuz K.D., Krishnan N.C. (2013). Transfer learning for activity recognition: A survey. Knowl. Inf. Syst..

[25] Inoue S., Pan X. Supervised and unsupervised transfer learning for activity recognition from simple in-home sensors. Proceedings of the 13th International Conference on Mobile and Ubiquitous Systems: Computing, Networking and Services.

[26] Wang W., Zheng V.W., Yu H., Miao C. (2019). A survey of zero-shot learning: Settings, methods, and applications. ACM Trans. Intell. Syst. Technol..

[27] Mikolov T., Sutskever I., Chen K., Corrado G.S., Dean J. (2013). Distributed representations of words and phrases and their compositionality. Advances in Neural Information Processing Systems.

[28] Mikolov T., Chen K., Corrado G., Dean J. (2013). Efficient estimation of word representations in vector space. arXiv.

[29] Pennington J., Socher R., Manning C. Glove: Global vectors for word representation. Proceedings of the 2014 Conference on Empirical Methods in Natural Language Processing (EMNLP).

[30] Cheng H.T., Sun F.T., Griss M., Davis P., Li J., You D. Nuactiv: Recognizing unseen new activities using semantic attribute-based learning. Proceedings of the 11th Annual International Conference on Mobile Systems, Applications, and Services.

[31] Cheng H.T., Griss M., Davis P., Li J., You D. Towards zero-shot learning for human activity recognition using semantic attribute sequence model. Proceedings of the 2013 ACM International Joint Conference on Pervasive and Ubiquitous Computing.

[32] Roggen D., Calatroni A., Rossi M., Holleczek T., Förster K., Tröster G., Lukowicz P., Bannach D., Pirkl G., Ferscha A. Collecting complex activity datasets in highly rich networked sensor environments. Proceedings of the 2010 Seventh International Conference on Networked Sensing Systems (INSS).

[33] Hammerla N.Y., Halloran S., Plötz T. (2016). Deep, convolutional, and recurrent models for human activity recognition using wearables. arXiv.

[34] Reiss A., Stricker D. Introducing a new benchmarked dataset for activity monitoring. Proceedings of the 2012 16th International Symposium on Wearable Computers.

[35] Kawaguchi N., Ogawa N., Iwasaki Y., Kaji K., Terada T., Murao K., Inoue S., Kawahara Y., Sumi Y., Nishio N. HASC Challenge: gathering large scale human activity corpus for the real-world activity understandings. Proceedings of the 2nd Augmented Human International Conference.

